# Uptake, distribution, clearance, and toxicity of iron oxide nanoparticles with different sizes and coatings

**DOI:** 10.1038/s41598-018-19628-z

**Published:** 2018-02-01

**Authors:** Qiyi Feng, Yanping Liu, Jian Huang, Ke Chen, Jinxing Huang, Kai Xiao

**Affiliations:** 1 National Chengdu Center for Safety Evaluation of Drugs, State Key Laboratory of Biotherapy, Collaborative Innovation Center for Biotherapy, West China Hospital, Sichuan University, Chengdu, China; 2 Safety Evaluation Center, Sichuan Institute for Food and Drug Control, Chengdu, China; 3 Department of Thoracic Surgery, Wuxi People’s Hospital of Nanjing Medical University, Wuxi, China; 4 Laboratory of Non-Human Primate Disease Model research, State Key Laboratory of Biotherapy, Collaborative Innovation Center for Biotherapy, West China Hospital, Sichuan University, Chengdu, China; 5 Department of Thoracic Surgery, West China Hospital, Sichuan University, Chengdu, China

## Abstract

Iron oxide nanoparticles (IONPs) have been increasingly used in biomedical applications, but the comprehensive understanding of their interactions with biological systems is relatively limited. In this study, we systematically investigated the *in vitro* cell uptake, cytotoxicity, *in vivo* distribution, clearance and toxicity of commercially available and well-characterized IONPs with different sizes and coatings. Polyethylenimine (PEI)-coated IONPs exhibited significantly higher uptake than PEGylated ones in both macrophages and cancer cells, and caused severe cytotoxicity through multiple mechanisms such as ROS production and apoptosis. 10 nm PEGylated IONPs showed higher cellular uptake than 30 nm ones, and were slightly cytotoxic only at high concentrations. Interestingly, PEGylated IONPs but not PEI-coated IONPs were able to induce autophagy, which may play a protective role against the cytotoxicity of IONPs. Biodistribution studies demonstrated that all the IONPs tended to distribute in the liver and spleen, and the biodegradation and clearance of PEGylated IONPs in these tissues were relatively slow (>2 weeks). Among them, 10 nm PEGylated IONPs achieved the highest tumor uptake. No obvious toxicity was found for PEGylated IONPs in BALB/c mice, whereas PEI-coated IONPs exhibited dose-dependent lethal toxicity. Therefore, it is crucial to consider the size and coating properties of IONPs in their applications.

## Introduction

Magnetic iron oxide nanoparticles (IONPs) have been used for a wide range of biomedical applications such as drug delivery, magnetic resonance imaging (MRI), thermal ablation therapy, *in vivo* cell tracking, and magnetic separation of cells or molecules^1^. In recent years, more and more nanomedicines have been already approved by the U.S. Food and Drug Administration (FDA) for human use, and some others are undergoing clinical trials^2^. For example, Feridex^®^ (ferumoxides) is the dextran-coated IONPs approved as a imaging contrast agent for the detection of liver lesions^3^. Feraheme^®^ (ferumoxytol) has been approved for the treatment of iron deficiency anemia in adult patients with chronic kidney disease^4^. The increasing applications have raised public concerns about the biosafety, long-term distribution, and clearance of IONPs.

Most IONPs introduced to the bloodstream are usually subjected to opsonization (adsorption of plasma proteins on the particles surface), followed by subsequent recognition and uptake by macrophages residing in the organs of the mononuclear phagocytic system (MPS), ultimately resulting in the elimination from the blood circulation^5^. It is generally believed that the interactions with biological components (e.g., proteins), cellular uptake, *in vivo* fate and toxicity of IONPs are strongly correlated with their physicochemical characteristics. For example, hydrodynamic size is one of the most important factors in determining the distribution and clearance of IONPs. It has been reported that IONPs larger than 100 nm in diameter are rapidly trapped in the liver and spleen through macrophage phagocytosis, whereas IONPs smaller than 10 nm in diameter are likely to be eliminated through renal clearance^6^. Ultrasmall super paramagnetic IONPs (<50 nm) is thought to benefit from slower opsonization and clearance from the reticuloendothelial system (RES)^7^. Besides, the size uniformity of IONPs will also affect their *in vivo* pharmacokinetics and biodistribution results, and a low polydispersity index (PDI) might be more desirable for uniform and repeatable performance. Surface coating is another important factor affecting the destiny and biological effects of IONPs. Due to the colloidal instability of bare IONPs, different types of natural and synthetic coating materials such as dextran, Pluronic, and polyethylene glycol (PEG) were used to improve the stability and blood circulation of IONPs. Among them, PEG is the most popular coating polymer, which has excellent anti-fouling property (preventing opsonization) and high steric hindrance to stabilize IONPs^8^. Some other types of capping agents are also used to coat the surface of IONPs for certain biomedical applications. For example, poly(ethylenimine) (PEI) is a cationic macromolecule commonly used in gene transfer/therapy protocols with high transfection efficiency both *in vitro* and *in vivo*^9^. When capped with amphiphilic PEI, the modified IONPs can capture negatively charged molecules such as DNA or RNA through charge-charge interaction, and may be used as MRI visible gene/drug delivery carriers and cell tracking probes^10^. The functional groups of coating materials presented on IONPs may also alter their surface charge accordingly, which will influence the protein absorption and subsequent biological behaviors of nanoparticles. For instance, Xiao *et al*. demonstrated that highly charged micellar nanoparticles (either positive or negative) were massively incorporated by macrophages in the liver, whereas nanoparticles with slightly negative charge exhibited the lowest macrophage clearance, and the highest tumor uptake^11^.

Although there are an increasing number of studies devoted to understanding the interaction of IONPs with biological systems, published data on the macrophage clearance, biodistribution and toxicity of IONPs are not consistent, which might be due to variation in IONPs characteristics (e.g., size, shape, surface coating, and surface charge) and experimental conditions (e.g., cell types, animal models, administration route, and quantification techniques) used in different studies^6^. Most of the cell studies focused on the uptake and cytotoxicity of IONPs in macrophages, with little attention paid to other non-phagocytic cells. Meanwhile, the specific mechanism of distinct cellular response caused by various IONPs is not yet clear. Furthermore, the distribution, degradation, clearance, and toxicity of IONPs with different physicochemical characteristics have not been determined in detail.

The aim of the present study is to investigate the influence of particle size and surface coating on the *in vitro* and *in vivo* biological behaviors of IONPs. Commercially available IONPs with different core size (10 nm or 30 nm) and surface coating (PEG or PEI) were used in this study. The uptake, intracellular localization, and cytotoxicity of these IONPs with different sizes and coatings were examined in both RAW264.7 macrophages and non-phagocytic SKOV-3 ovarian cancer cells. Next, the potential cytotoxic mechanisms of these IONPs were explored. Finally, the *in vivo* biodistribution, tumor uptake, clearance, and toxicity of these IONPs were investigated in both SKOV-3 tumor bearing nude mice and BALB/c mice, respectively.

## Results

### Particle characterization

**Figure 1:**
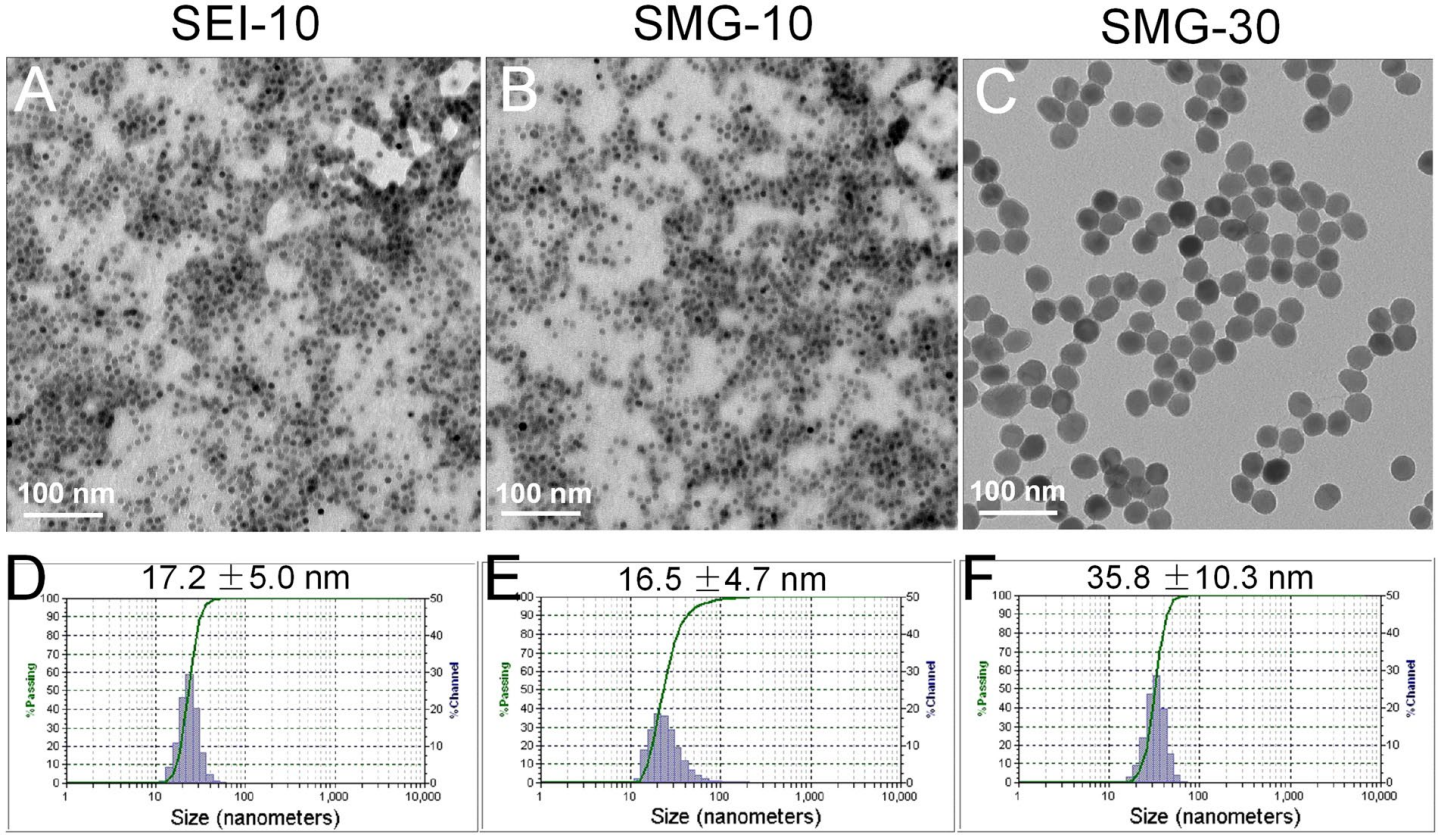
Representative TEM images (**A,B,C**) and dynamic light scanning (DLS) size distribution (**D,E,F**) of SEI-10, SMG-10, and SMG-30.

The morphology and particle size of IONPs were observed under TEM. As shown in Fig. 1A,B and C, all these IONPs were spherical and homogeneous, and the core sizes of SEI-10, SMG-10 and SMG-30 were very close to their theoretical values (10 nm, 10 nm, and 30 nm in diameter) stated by the manufacture. According to the information provided by the manufacture, the hydrodynamic size of IONPs is about 5–10 nm larger than their inorganic core size measured by TEM, due to the attachment of organic layers (PEG or PEI). DLS measurement (Fig. 1D,E and F) demonstrated that the hydrodynamic size of SEI-10, SMG-10 and SMG-30 were 17.2 ± 5.0 nm, 16.5 ± 4.7 nm, and 35.8 ± 10.3 nm, respectively. The polydispersity index (PDI) of SEI-10, SMG-10 and SMG-30 were 0.192, 0.163, and 0.105, respectively, and their zeta potential were +29.28 mV, −0.52 mV, and −0.52 mV, respectively (Table 1). There was no apparent aggregation when these IONPs were incubated with Borate, Tris, HEPES or PBS buffer (data not shown), indicating that they were stable in the buffer solution. To predict the colloidal stability of IONPs *in vivo*, their size distribution in the presence of human plasma (50% in volume) was monitored by DLS measurement over time. As shown in Supplemental Fig. 1A, the particle size of SMG-10 and SMG-30 in human plasma were uniform during the first 24 h, although a small portion of aggregates was found at 48 h. In contrast, SEI-10 displayed scattered size distribution after mixing with human plasma, and more than 50% population had the average size of thousands of nanometers. Obvious precipitation was found in SEI-10 plasma samples, but not in SMG-10 and SMG-30 samples at 48 h (Supplemental Fig. 1B). These results suggest that SMG-10 and SMG-30 were relatively stable in *in vitro* cell culture medium and *in vivo* blood circulation, and SEI-10 tended to form large aggregates in such conditions.

**Table 1:** The physicochemical characteristics of different IONPs.

IONPs	Size by TEM (nm)	Hydrodynamic size by DLS (nm)	Polydispersity index (PDI)	Zeta potential (mV)
SEI-10	10	17.2 ± 5.0	0.192	+29.28
SMG-10	10	16.5 ± 4.7	0.163	−0.52
SMG-30	30	35.8 ± 10.3	0.105	−0.52

### Particle uptake and localization

**Figure 2:**
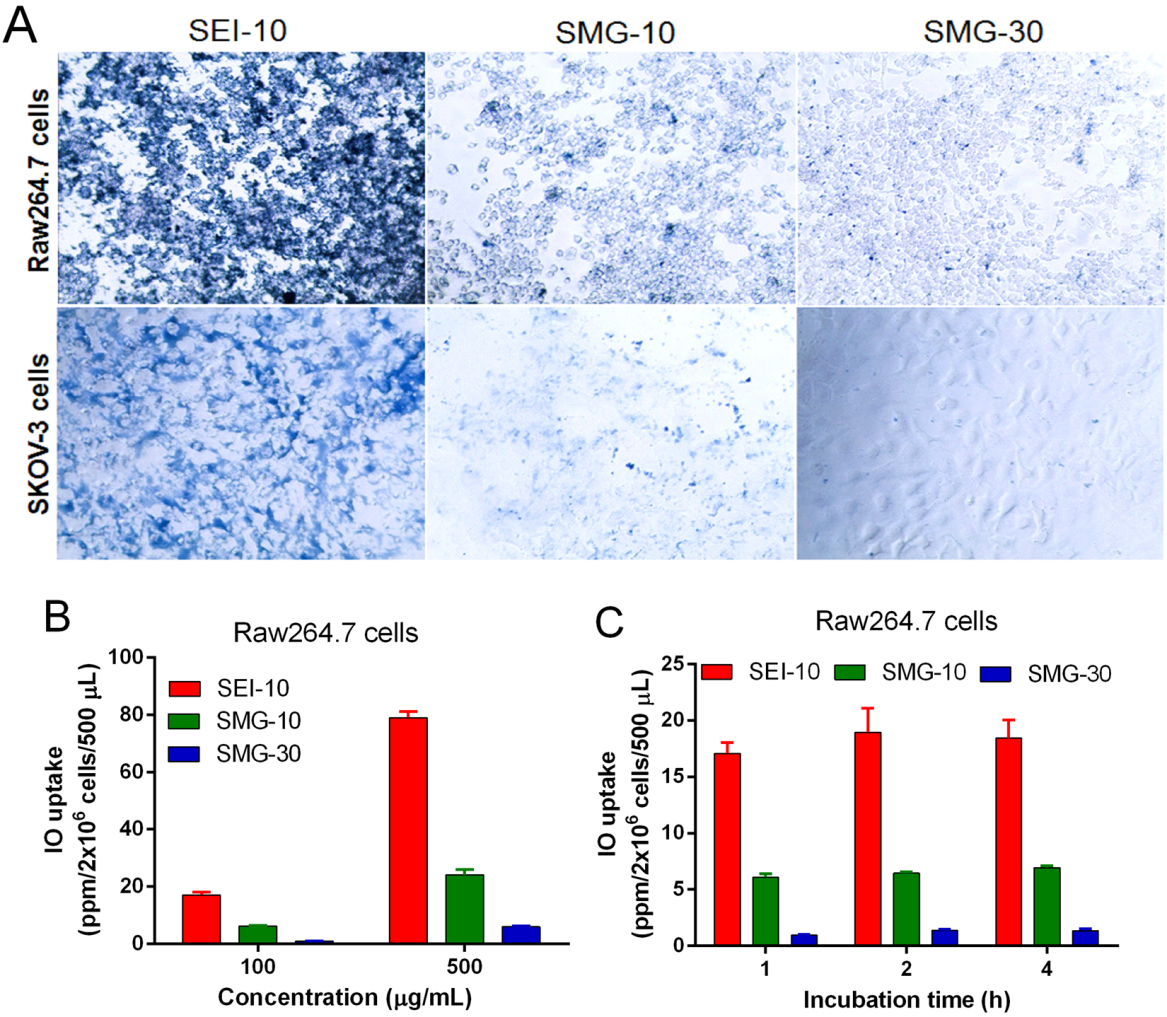
Cell uptake of different IONPs in RAW264.7 macrophage cells and SKOV-3 cells. (**A**) Prussian blue staining of iron in RAW264.7 cells and SKOV-3 cells after incubation with SEI-10, SMG-10, and SMG-30 at a concentration of 200 μg/mL for 4 h, respectively. Magnification, 20×. ICP-MS measurement of iron concentrations in RAW264.7 cells after incubation with IONPs at different concentrations for 1 h (**B**) or at a concentration of 100 μg/mL for different period of time (**C**).

**Figure 3:**
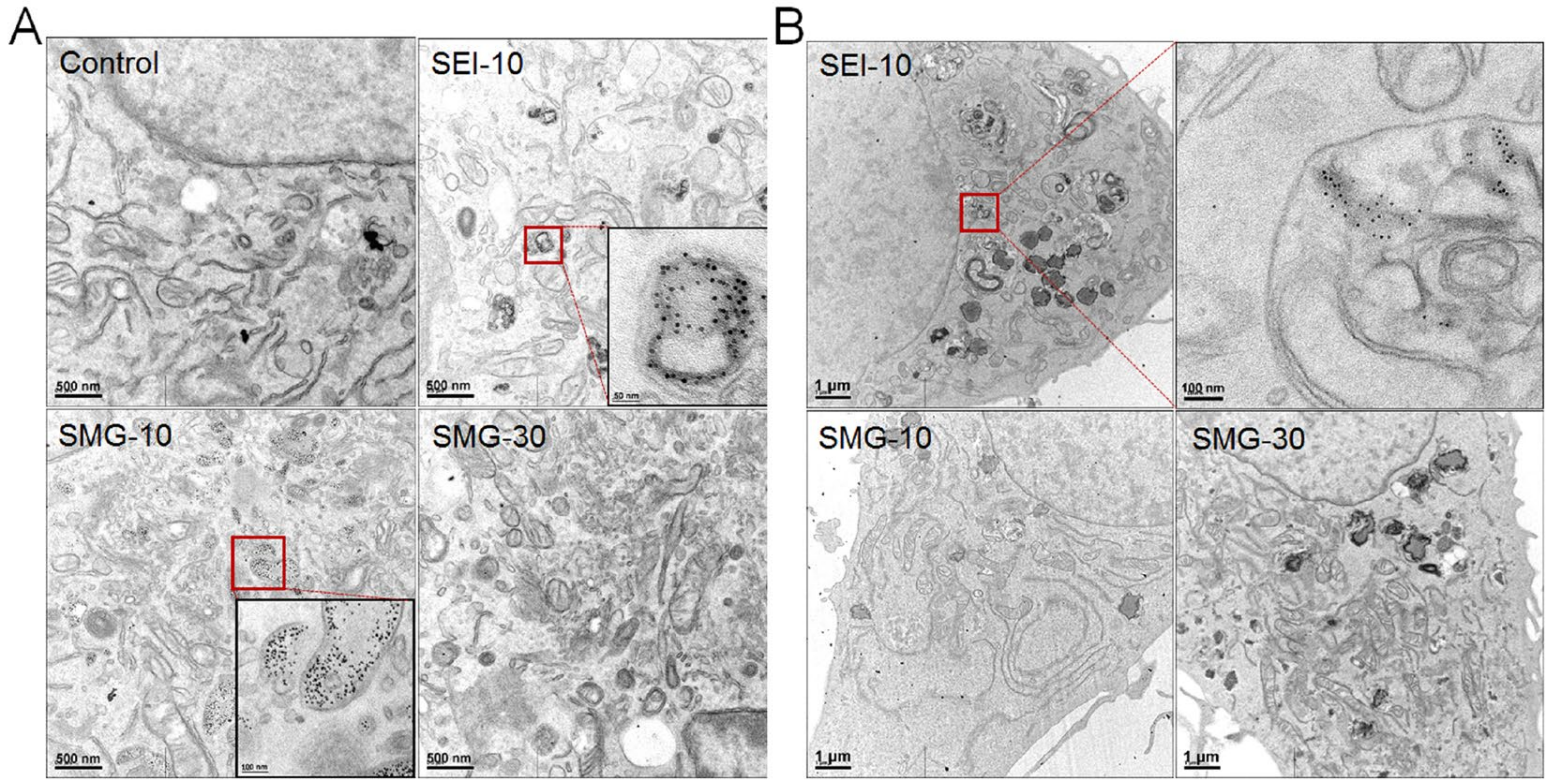
TEM images of RAW264.7 macrophages (**A**) and SKOV-3 cells (**B**) after 2-h exposure of different IONPs at the concentration of 100 µg/mL.

The uptake of IONPs in RAW264.7 macrophages and SKOV-3 cells were qualitatively visualized by Prussian blue staining (Fig. 2A). The results demonstrated that SEI-10 had higher uptake, shown as more blue deposition, than SMG-10 and SMG-30 in both macrophages and cancer cells after 4-h incubation. In addition, the uptake of these IONPs in RAW264.7 macrophages appeared to be higher than that in SKOV-3 cancer cells. The particles uptake in macrophages was further quantitatively measured by ICP-MS. As shown in Fig. 2B, IONPs were internalized in macrophages in a concentration-dependent manner. Among these IONPs, SEI-10 had the highest cellular uptake in macrophages, followed by SMG-10, and finally SMG-30, which was consistent with Prussian blue staining results. No proportional increase of cellular uptake was found for all these IONPs when the exposure time increased from 1 h to 4 h (Fig. 2C), indicating the rapid phagocytosis of IONPs by macrophages. Intracellular particle localization of IONPs upon their uptake was further investigated using TEM images. After 2-h exposure, a large number of individually dispersed SEI-10, less SMG-10 and little SMG-30 were internalized and mainly localized within membrane-bound structures (*e.g*., endosome, lysosome) in both RAW264.7 macrophages and SKOV-3 cancer cells (Fig. 3). Interestingly, it appeared that the uptake of SEI-10 occurred after membrane accumulation (Supplemental Fig. 4), suggesting that the adsorptive endocytosis is the main mechanism of cell uptake of positively charged SEI. However, PEG-coated IONPs were independent from membrane accumulation, and seemed to follow common endocytic internalization processes and receptor-mediated endocytosis^12^. In addition, no signs of nuclear localization were found for all these IONPs.

### Cytotoxicity assays

**Figure 4:**
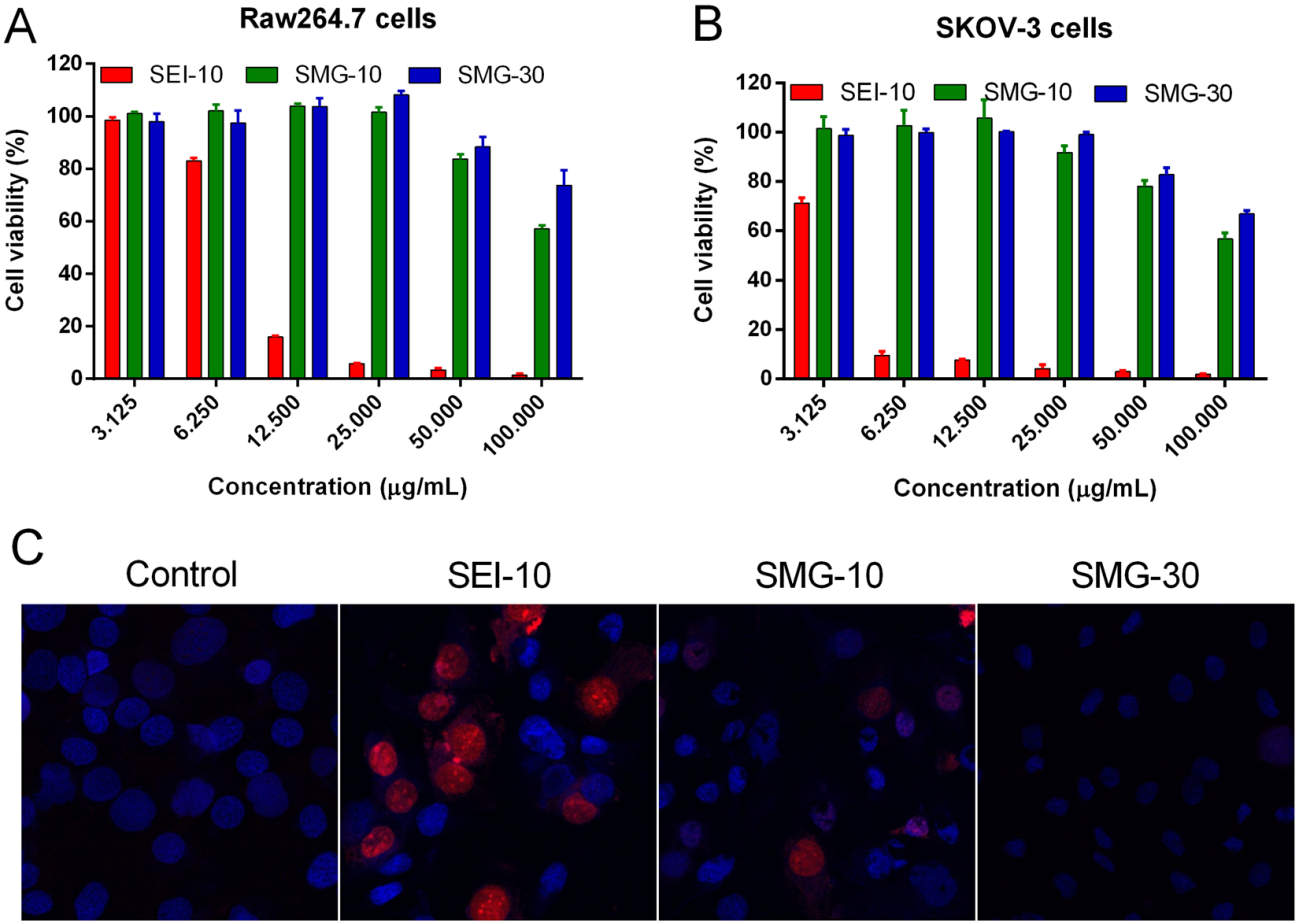
The *in vitro* cytotoxicity of different IONPs. The cell viability of RAW264.7 macrophages (**A**) and SKOV-3 cells (**B**) after 48-h treatment of IONPs with different concentrations were measured by MTS assay. (**C**) Representative fluorescent microscopic images showing the cell death mode induced by various IONPs in SKOV-3 cells. The cells were treated with SEI-10 (5 µg/mL), SMG-10 (400 µg/mL), or SMG-30 (400 µg/mL) for 16 h, and then stained with Hoechst 33342 (blue) and PI (red).

As illustrated in Fig. 4A and B, SEI-10 induced dose-dependent cytotoxicity against both RAW264.7 macrophages and SKOV-3 cancer cells at the test concentrations (3.125–100 µg/mL), and SKOV-3 cells were relatively more susceptible to SEI-10 toxicity than RAW264.7 macrophages. In contrast, no appreciable cytotoxic effects were observed for both SMG-10 and SMG-30 at the concentration of 25 µg/mL and below in both cells, although slight cytotoxicity was shown at the concentration above 50 µg/mL. The cell death induced by IONPs was further confirmed by Hoechst 33342 and PI staining. Hoechst 33342 stains the chromatin in living cells while PI is only permeable to dead cells. After 16-h exposure to 5 µg/mL SEI-10, the majority of nuclei in SKOV-3 cells displayed positive PI staining, which was the sign of cell death (Fig. 4C). In contrast, only a small portion of nuclei of cells treated with SMG-10 showed positive PI staining at the extremely high concentration (400 µg/mL), and the PI staining of cells treated with SMG-30 was almost negative even at the concentration up to 400 µg/mL.

### Mechanisms underlying the cytotoxicity of IONPs

#### Cell membrane integrity

**Figure 5:**
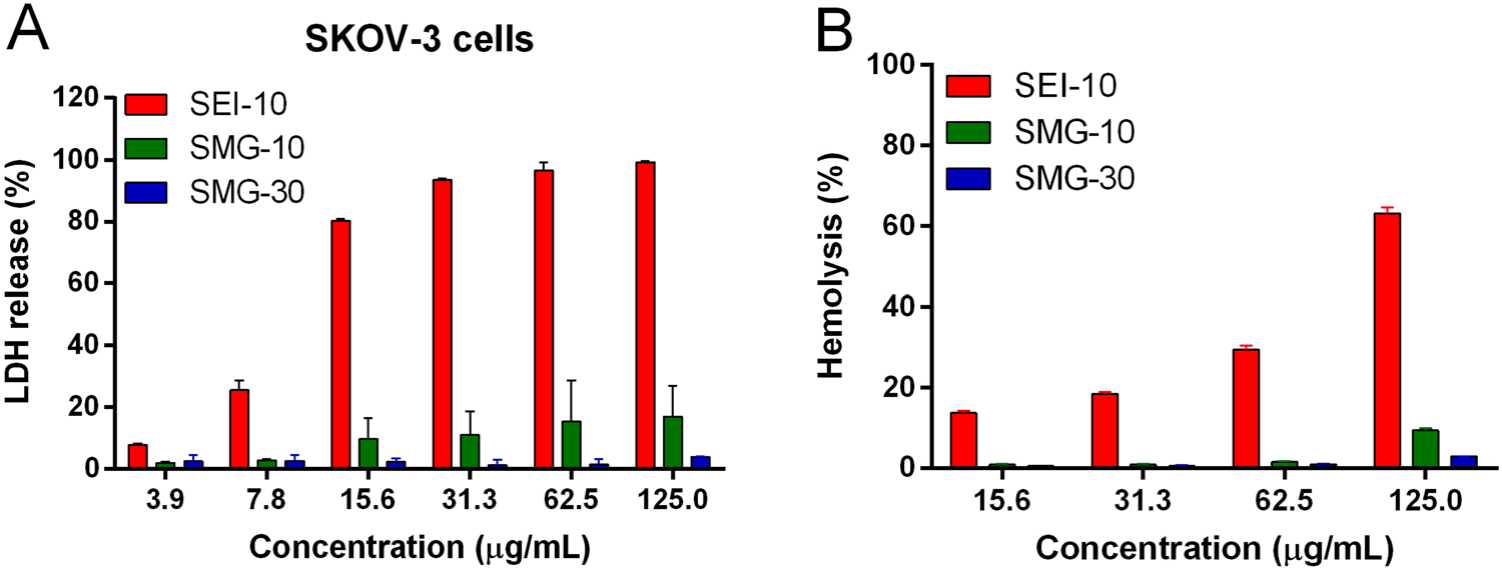
The LDH toxicities and hemolytic activities of different IONPs. (**A**) LDH release in SKOV-3 cells incubated with different concentrations of IONPs for 4 h. (**B**) *In vitro* red blood cells (RBCs) lysis. IONPs were incubated with mouse erythrocyte suspension for 4 h at 37 °C. RBCs lysis was determined spectrophotometrically (Absorbance 540 nm) based on hemoglobin level. PBS was used as negative control, and Triton-100 was used as positive control. Data represent mean ± SEM (n = 3).

The effect of IONPs on the cell membrane integrity was evaluated by LDH assay, which determines the amount of LDH enzyme released into the culture medium. As shown in Fig. 5A, SEI-10 treatment led to the dose-dependent release of LDH in SKOV-3 cells in at concentrations ranging from3.9 to 125 µg/mL. In contrast, only minimal or negligible LDH release was found in cells treated with SMG-10 or SMG-30 at the tested concentrations.

The hemolytic properties of IONPs were evaluated in mouse erythrocyte suspension after 4-h incubation at 37 °C. The results (Fig. 5B) demonstrated that both SMG-10 and SMG-30 had almost no hemolytic activity at the concentrations ranging from 15.6 to 125 µg/mL, whereas SEI-10 showed severe dose-dependent hemolysis.

#### Apoptosis

**Figure 6:**
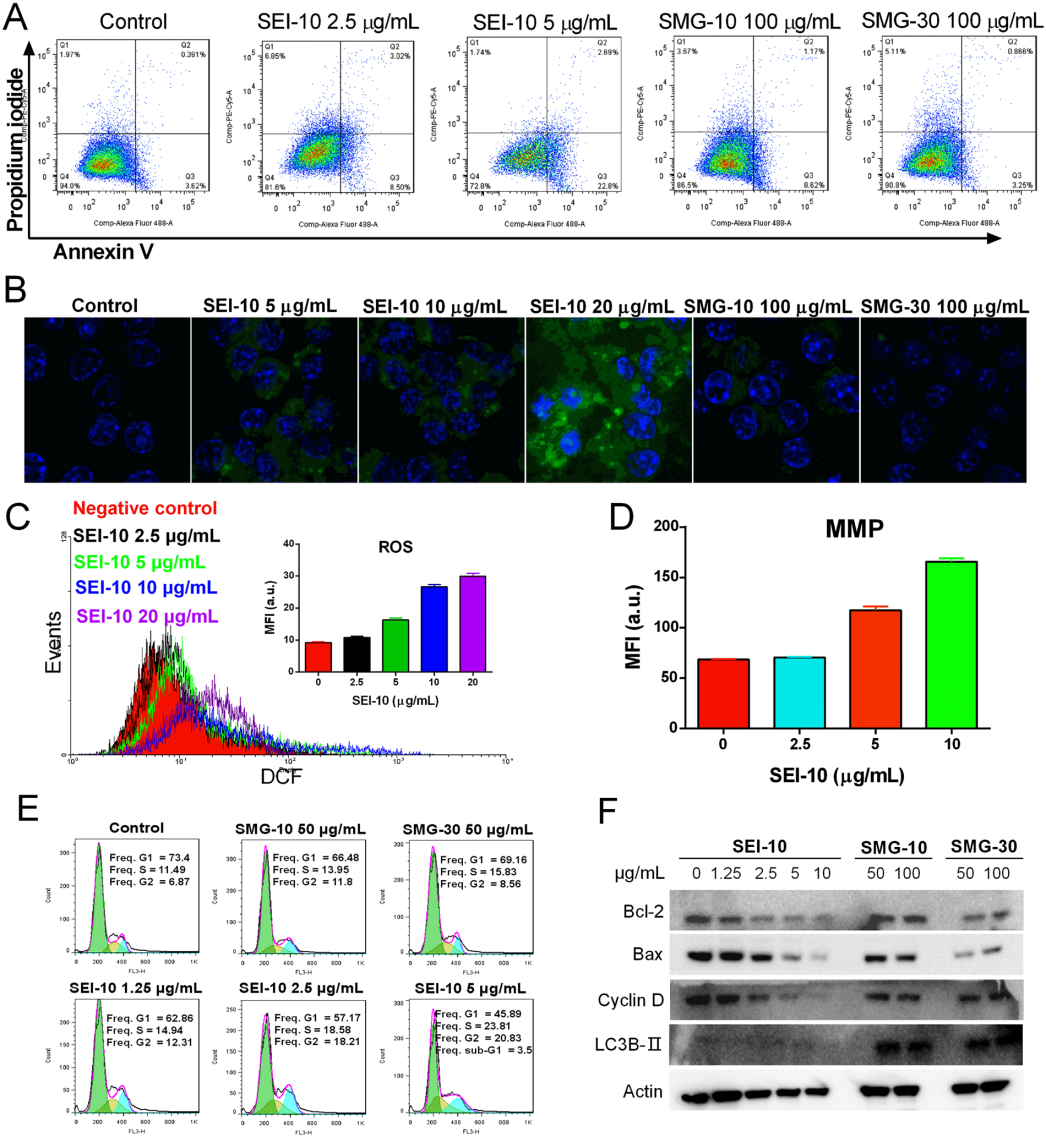
The potential mechanisms underlying the cell death induced by IONPs in SKOV-3 cells. (**A**) Apoptotic and necrotic cell death were analyzed by Annexin V and PI dual staining after IONPs treatment for 24 h in SKOV-3 cells. Intracellular reactive oxygen species (ROS) production in SKOV-3 cells treated with various IONPs for 18 h was detected using a H_2_DCFDA probe by confocal microscopy (**B**) and flow cytometry (**C**), respectively. (**D**) The measurement of membrane mitochondria potential (MMP) of SKOV-3 cells treated with different concentrations of SEI-10 for 18 h. (**E**) the effect of IONPs on the cell cycle in SKOV-3 cells after 24-h treatment. (**F**) The effects of IONPs on the expression of proteins involved in apoptosis (Bcl-2, Bax), cell cycle (Cyclin D), and autophagy (LC3B-II), determined by western blot analysis after 18-h treatment.

Apoptotic or necrotic cell death was discriminated by Annexin V/PI dual staining. After 24-h incubation, SEI-10 induced dose-dependent apoptosis in both SKOV-3 cells (Fig. 6A) and RAW264.7 macrophages (Supplemental Fig. 2A). For example, the total population of early apoptosis (Q3) and late apoptosis (Q2) in SKOV-3 cells treated with PBS control, 2.5 µg/mL SEI-10, and 5 µg/mL SEI-10 were 4.01%, 11.52%, and 25.49%, respectively. In contrast, only mild apoptosis was observed in cells treated with SMG-10 and SMG-30 at extremely high concentration (100 µg/mL).

#### ROS production

The intracellular ROS production after IONPs exposure was measured using a DCF-DA fluorescent probe. Fluorescence microscopic images (Fig. 6B, Supplemental Fig. 2B) demonstrated that SMG-10 and SMG-30 did not induce obvious ROS formation in both SKOV-3 cells and RAW264.7 macrophages after 18-h exposure at the concentration up to 100 μg/mL, whereas SEI-10 significantly increased the production of ROS at the concentrations ranging from 5 to 20 μg/mL, when compared to the unexposed cells. Flow cytometric analysis (Fig. 6C, Supplemental Figs 2C and 3) further quantitatively confirmed that SEI-10, but not SMG-10 and SMG-30, induced the dose-dependent enhancement of intracellular ROS generation in both SKOV-3 and RAW264.7 cells.

#### MMP

Since mitochondria might be impaired by the over production of ROS, the effect of SEI-10 on the MMP in cells was measured with DiOC6 dye staining followed by flow cytometric analysis. The results demonstrated that SEI-10 exposure (0–10 μg/mL) induced a concentration-dependent increase of green fluorescence intensity in SKOV-3 cells (Fig. 6D) and RAW264.7 macrophages (Supplemental Fig. 2D), indicating a loss of MMP in treated cells.

#### Cell cycle

As demonstrated in Fig. 6E, SMG-10 and SMG-30 did not affect the cell cycle in SKOV-3 cells at the concentration up to 50 μg/mL. On the contrary, SEI-10 at concentrations ranging from 1.25 to 5 μg/mL induced a dose-dependent increase in the percentage of cells in the G2 phase with a concomitant decrease in G1 phase. In addition, the sub G1 population (cell fragments) increased after the exposure of 5 μg/mL SEI-10, which is a marker of apoptosis.

#### Western blot

It was observed that the levels of anti-apoptotic protein Bcl-2 and pro-apoptotic protein Bax were down-regulated in SKOV-3 cells treated with SEI-10 in a dose-dependent manner when compared with control group, but there was no obvious difference in the Bcl-2/Bax ratio. Meanwhile, SEI-10 treatment resulted in a dose-dependent decrease in the protein expression of cyclin D, which is involved in the cell cycle regulation (Fig. 6F). Interestingly, the treatment of cells with SMG-10 and SMG-30, but not SEI-10, significantly increased the expression of autophagy marker LC3B-II (2–3 fold increase, Supplemental Fig. 5). To confirm the autophagy formation induced by PEGylated IONPs, TEM imaging was further performed to identify autophagosomes in IONPs treated cells. As shown in Supplemental Fig. 6, double-or multiple-membraned autophagosomes were observed in cells treated with SMG-10.

#### *In vivo* biodistribution

**Figure 7:**
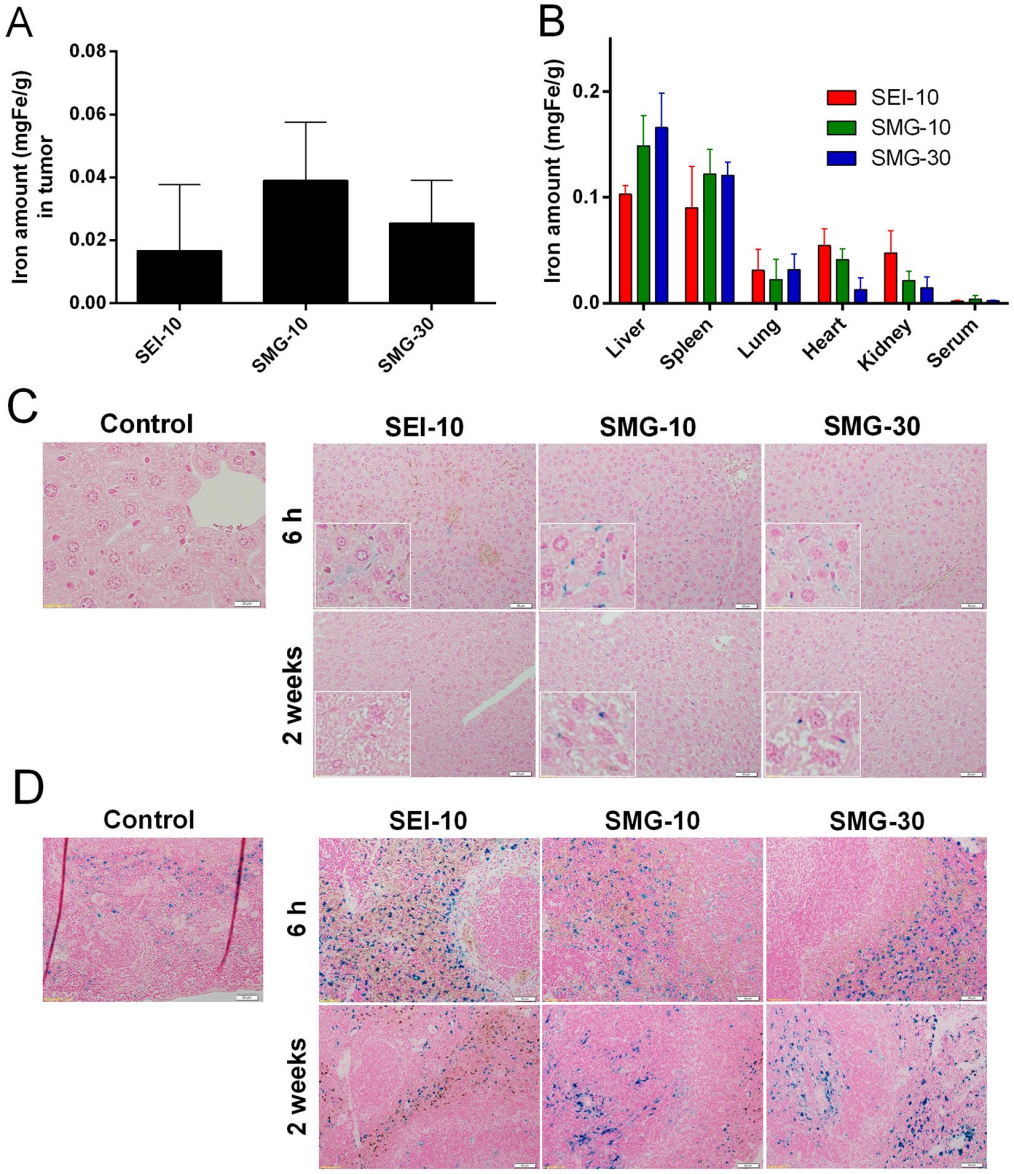
*In vivo* biodistribution and clearance of different IONPs (1.5 mgFe/kg) in SKOV-3 tumor bearing mice after intravenous injection. The distribution of IONPs in the tumors (**A**) and other indicated organs (**B**) were quantified by ICP-MS at 24 h post-injection. Histological sections showing distribution, degradation, and clearance of IONPs in the liver (**C**) and spleen (**D**). At 6 h and 2 weeks post-injection, the sections of liver and spleen tissues harvested from the mice were processed with Prussian blue and nuclear fast red staining. The control samples were tissues from animals that were not injected with IONPs. The insets in (**C**) indicate the magnified images showing the Kupffer cells.

We next investigated the *in vivo* accumulation, biodistribution, degradation, and clearance of the IONP formulations in SKOV-3 tumor bearing mice. Excess iron distributed in the tumors obtained at 24 h post-injection using ICP-MS measurement, demonstrated that all the IONPs were able to accumulate in the tumor sites to varying extent *via* the enhanced permeability and retention (EPR) effects, and the tendency was: SMG-10 > SMG-30 > SEI-10 (Fig. 7A). Besides, these IONPs tended to be trapped in the MPS organs such as liver and spleen (Fig. 7B), with a little less amount distributed in other organs such as lung, heart, and kidney. Interestingly, the uptake of SEI-10 in the kidney was significantly higher than that of SMG-10 and SMG-30, indicating its faster clearance. At 24 h post-injection, the iron concentration in the serum was almost undetectable.

Tissue samples dissected at different time points (6 h and 2 weeks) after administration were stained with Prussian blue (iron) to observe time-dependent degradation and clearance of the IONPs accumulated in the liver and spleen (Fig. 7C,D). In particular, most of the IONPs were found in the Kupffer cells located in the linings of the hepatic sinusoids, as shown in the enlarged images. Consistent with the ICP-MS assay results, the iron staining of SEI-10 in the hepatic tissues at 6 h post-injection appeared weaker than that of SMG-10 and SMG-30. At 2 weeks post-injection, almost no staining was observed in the liver tissue of mice injected with SEI-10, whereas significant staining remained at the liver tissue of animals injected with SMG-10 or SMG-30. In the spleen, substantial iron staining was found in the tissue samples for all the IONPs formulations at 6 h post-injection. At 2 weeks post-injection, only minimal iron staining was left in the spleen tissue samples of SEI-10, whereas the majority of iron staining signals still remained in those of SMG-10 and SMG-30.

#### *In vivo* toxicity

**Figure 8:**
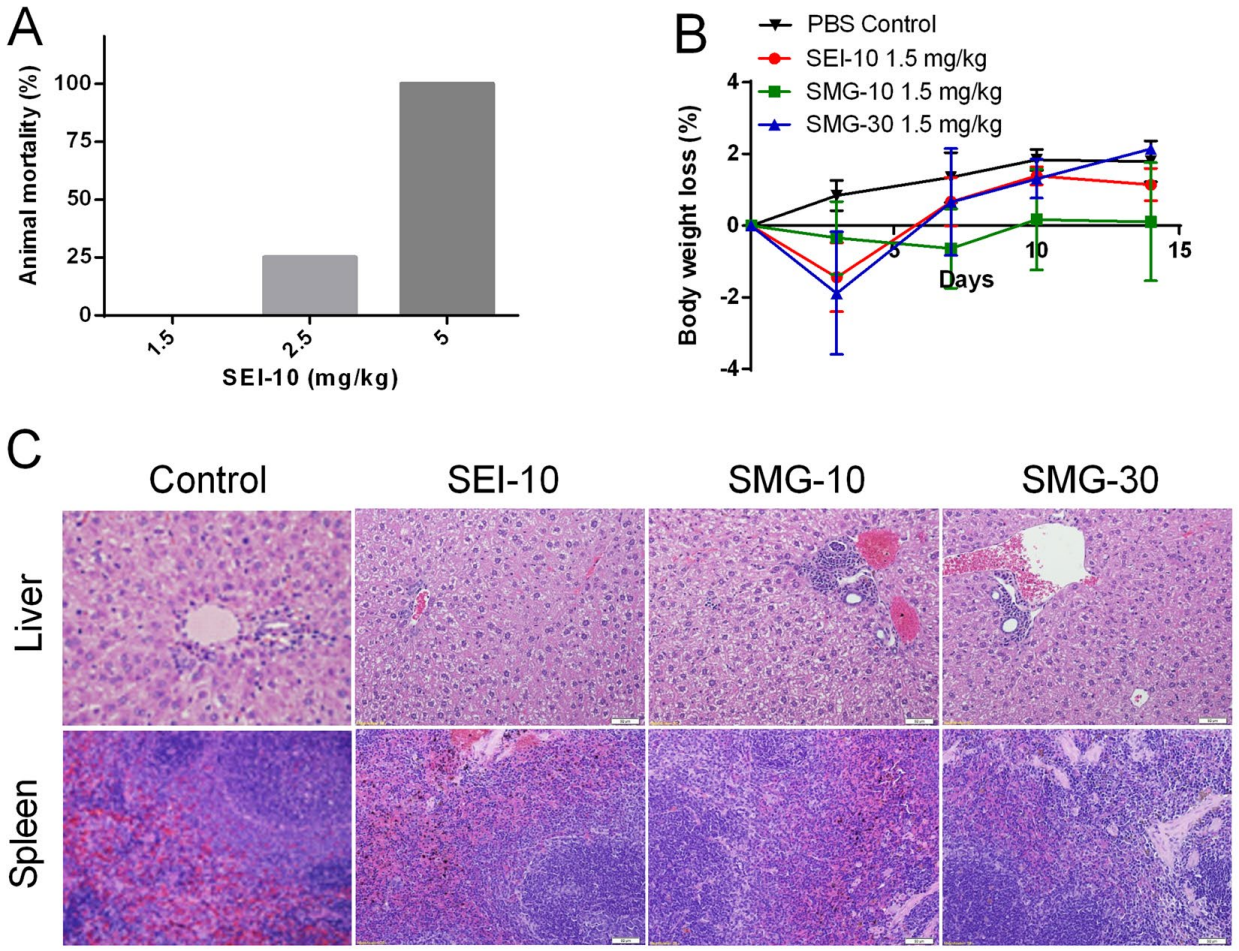
*In vivo* toxicity of different IONPs in BALB/c mice. (**A**) The mortality of BALB/c mice (n = 4) receiving different doses of SEI-10. (**B**) The body weight change of mice injected with various IONPs at the dose of 1.5 mg/kg, compared with PBS control. (**C**) Representative histopathological images of liver and spleen. Livers and spleens were harvested from mice on 2 weeks after intravenous injection of various IONPs, and stained with hematoxylin and eosin. Slight mononuclear cell infiltration in the portal area of the liver could be identified in mice with SMG-10 and SMG-30. Splenic plasmacytosis was also noted in mice treated with SMG-30, which may represent a response to antigenic stimulation.

In the preliminary study, all mice in 5 mg/kg SEI-10 group and a quarter of mice in 2.5 mg/kg SEI-10 died within 24 h post-injection (Fig. 8A), and the maximum tolerated dose (MTD) of SEI-10 in BALB/c mice was approximately 1.5 mg/kg. In contrast, no animal deaths were found for SMG-10 and SMG-30 at dose up to 5 mg/kg (data not shown). Since we observed significant amount of PEGylated IONPs remaining in the liver and spleen two weeks post-injection, we searched for signs of *in vivo* toxicity by monitoring body weight, hematology and blood biochemical indexes. The mice in all the IONPs treatment groups underwent transient slight body weight loss after administration (1.5 mg/kg), but they recovered gradually during one week (Fig. 8B), and no signs of distress such as behavioral changes were observed. The hematology (Supplemental Table 1) and blood chemistry (Supplemental Table 2) results on day 7 post-injection showed that blood cell counts and four indexes (AST, total bilirubin, BUN, and creatinine) out of the five hepatic and renal function panels were within the normal range, except that the level of ALT enzyme in mice treated with SMG-10 slightly increased compared to PBS control. On day 14 post-injection, the increased ALT level in SMG-10 treated mice returned to normal, and all other serum chemistry indexes were also in the normal ranges (data not shown). At 2 weeks post-injection, the liver and spleen tissues were harvested for histopathological examination. As shown in Fig. 8C, slight mononuclear cell infiltration in the portal area of the liver was identified in mice treated with SMG-10 and SMG-30. Splenic plasmacytosis was also noted in mice treated with SMG-30, which may represent a response to antigenic stimulation, presumably due to the uptake of exogenous IONPs.

## Discussion

It is generally believed that the size of nanoparticles for cancer therapy should be in the range of 5–100 nm, in which the blood circulation time and EPR effect are maximized^13–16^. If the particle size is too small, it can be rapidly cleared through the kidneys, but if too large, it is efficiently trapped by cells in the reticuloendothelial system (RES) organs^17^. Meanwhile, appropriate surface coating is essential for biomedical applications of IONPs. For example, PEG is usually used to prevent the aggregation and opsonization of nanoparticles, reduce the macrophage uptake, and increase the blood circulation time^18^; PEI is utilized to deliver DNA/siRNA due to its inherent ability to condense genetic material^19^. In this study, our aim was to understand the influence of particle size and surface coating on the biological distribution of IONPs and their biological effects *in vitro* and *in vivo*.

Commercially available IONPs with different particle sizes (10 nm or 30 nm) and surface coatings (PEG or PEI) were employed in the study. Their physicochemical properties including particle size, PDI, surface charge, and stability were well-characterized. After surface coating, the hydrodynamic sizes of SEI-10, SMG-10, and SMG-30 were approximately 17 nm, 17 nm, and 36 nm, respectively, and their PDI were less than 0.2, indicating their monodisperse distribution. The surface charge of IONPs with PEI coating (SEI-10) was positive, while IONPs with PEG coating (SMG-10 and SMG-30) were almost neutral. IONPs with PEI coating tended to rapidly form large aggregates in the presence of plasma, whereas PEG coating dramatically prevented the aggregation of IONPs, showing much better colloidal stability in the physiological condition.

In the *in vitro* cellular uptake studies, both Prussian blue staining and ICP-MS measurement demonstrated that PEI-coated IONPs were internalized more efficiently than PEGylated IONPs with the same particle size, which may be due to the affinity of cationic nanoparticles to the negative phospholipid head groups or protein domains on cellular membranes^20^. Besides, small PEGylated IONPs (10 nm) showed relatively higher cellular uptake than large ones (30 nm), which is consistent with other previously reported nanoparticles^7^. TEM observation demonstrated that all the IONPs were distributed primarily in endosomes and/or lysosomes in both macrophages and cancer cells upon their uptake. However, SEI-10 was likely to be internalized through adsorptive endocytosis, while PEGylated IONPs seemed to be internalized through common endocytic internalization processes and receptor-mediated endocytosis^12^. In addition, no particles were found in the nuclei of cells treated with all the IONPs. Therefore, the observed genotoxic effects (mutagenicity) in our previous studies are presumably not due to the direct interaction of particles with DNA in the nucleus of cells^21,22^. In the *in vitro* cytotoxicity study, PEI-coated IONPs exhibited severe cytotoxicity against both macrophages and cancer cells at low concentrations, while no obvious cytotoxicity was observed for PEGylated IONPs at even much higher concentrations. In addition, SKOV-3 cancer cells seemed to be more susceptible to the cytotoxicity of SEI-10 than RAW264.7 macrophages. Owing to its special immune characteristics, macrophages show specific phagocytic ability, and tend to engulf the exogenous IONPs as demonstrated in our study. On the other hand, it has been reported that macrophages are highly tolerant to the cytotoxicity of anticancer drugs and nanoparticles^23^. Multiple molecular mechanisms were found to be involved in the cytotoxicity of PEI-coated IONPs, including the disruption of cell membrane integrity, ROS generation, apoptosis, and G2-phase cell cycle arrest. We speculate that the cytotoxicity of PEI-coated IONPs may originate from the PEI coating material, which was reported to be cytotoxic *via* two different mechanisms including the disruption of the cell membrane leading to necrotic cell death, and disruption of the mitochondrial membrane after internalization leading to apoptosis^24^. To overcome the toxicity issue and improve the biocompatibility of PEI-coated IONPs in biomedical applications such as gene delivery and MRI imaging, various optimization strategies have been adopted. For example, researchers utilized lactose to modify amphiphilic low molecular weight polyethylenimine (C12-PEI2K) at different lactosylation degree and demonstrated that the lactose modification can considerably reduce the cytotoxicity of PEI-coated IONPs without compromising their labeling efficacy as well as MR imaging capability^25^. It is also reported serum proteins could mask the cationic PEI surface of IONPs in a dose-dependent manner, leading to concurrent incremental decreases in cationic IONPs cytotoxicity^26^. In addition, it was interesting to find that IONPs with PEG coating but not PEI coating were able to induce autophagy, which may play a protective role against the cytotoxicity of IONPs.

Because of the high affinity with plasma proteins and rapid clearance of macrophages, positively charged nanoparticles are generally considered to have faster blood clearance than neutral particles. As expected, SEI-10 showed the lowest uptake in tumor tissue, whereas SMG-10 achieved the highest tumor uptake, followed by SMG-30. Meanwhile, it was not surprising that IONPs, irrespective of size and charge, mainly accumulated in the RES such as liver and spleen, where most macrophages reside. Prussian blue staining of liver and spleen sections showed that the degradation and removal of PEI-coated IONPs was faster than that of PEGylated IONPs, and PEGylated IONPs remained in the liver and spleen after two weeks of administration. Previous studies have demonstrated that IONPs are typically metabolized in the liver and other MPS organs. They are degraded in various ferrous Fe(II) and ferric Fe(III) products, which will be instead recycled and eventually incorporated into either the storage or utilization pathways (e.g. hemoglobin, ferritin, and transferrin)^27^. Toxicity study demonstrated that SEI-10 at the dose of 2.5 mg/kg and above may lead to animal death, which was likely due to the rapid hemolysis or capillary blockage as a result of large aggregates formation. Transient increase of ALT enzyme and slight histopathological changes in the liver and spleen were found in mice treated with IONPs with PEG coating. Long-term *in vivo* toxicity of IONPs warrants further investigation in consideration of their slow rate of degradation and clearance.

In summary, we have demonstrated both size and coating have remarkable impact on the cellular uptake, cytotoxicity, distribution and clearance of INOPs. When compared with PEGylated IONPs, PEI-coated ones had higher cellular uptake, stronger cytotoxicity, faster clearance, and less tumor distribution. Among PEGylated INOPs, 10 nm ones exhibited relatively higher cellular uptake and tumor accumulation than 30 nm ones. Therefore, the critical effects of particle size and surface coating have to be carefully considered in the biomedical applications of IONPs in order to improve their biocompatibility, prevent their undesirable clearance, and facilitate their targeted delivery to the disease site.

## Materials and Methods

### Materials

IONPs with PEG (MW = 2 kDa) coating (10 nm, SMG-10, Catalog No. 031412; 30 nm, SMG-30, Catalog No. 020613)) and IONPs with PEI (MW = 25 kDa) coating (10 nm, SEI-10, Catalog No. 111910) were obtained from Ocean NanoTech, LLC (San Diego, CA, USA). 2′, 7′-Dichlorofluorescin diacetate (DCF-DA), Iron Assay Kit, and nuclear fast red solution were purchased from Sigma-Aldrich (St Louis, MO, USA). DiOC6(3) and Hoechst 33342 were purchased from Thermo Fisher Scientific (Waltham, MA, USA). Fluorescein isothiocyanate (FITC) Annexin V Apoptosis Detection Kit was purchased from BD Biosciences (San Jose, CA, USA). CytoTox 96^®^ Non-Radioactive Cytotoxicity Assay Kit (lactate dehydrogenase (LDH) detection kit) and CellTiter 96^®^ Aqueous Non-Radioactive Cell Proliferation Assay Kit (MTS assay kit) were purchased from Promega Corporation (Fitchburg, WI, USA). All antibodies were purchased from Cell Signaling Technology (Danvers, MA, USA).

### Particle characterization

The morphology of IONPs was observed on a transmission electron microscope (TEM, Philips CM-120) at an acceleration voltage of 80 kV. The hydrodynamic particle size and zeta potential of IONPs were measured by the dynamic light scattering (DLS) technique using Zetatrac (Microtrac) as described previously^11^. The stability of these NPs in 50% human plasma was evaluated by monitoring the particle size of NPs over time.

### Cell culture

SKOV-3 human ovarian cancer cells and RAW 264.7 murine macrophages were purchased from American Type Culture Collection (ATCC; Manassas, VA, USA), and cultured at 37 °C with 5% CO_2_ in McCoy’s 5a Medium and Dulbecco’s Modified Eagle’s Medium (DMEM) supplemented with 10% (v/v) fetal bovine serum, 100 U/mL penicillin G, and 100 mg/mL streptomycin, respectively.

### Animals

Specific pathogen-free healthy athymic nude mice (Nu/Nu strain) (6–8 weeks old) and BALB/c mice (5–7 weeks old) were obtained from Beijing Vital River Laboratory Animal Technology Co., Ltd (Beijing, China). The animals were housed in clean polypropylene cages and maintained in an air-conditioned conventional animal house at 23 ± 3 °C, 40–70% relative humidity and 12 h light/dark cycle. All animal experiments were conducted in accord with Association for Assessment and Accreditation of Laboratory Animal Care (AAALAC) guidelines, and protocols were approved by the Institutional Animal Care and Use Committee (IACUC) of National Chengdu Center for Safety Evaluation of Drugs, West China Hospital, Sichuan University.

### Particle uptake and localization

#### Prussian blue staining

RAW264.7 macrophages and SKOV-3 cells were incubated with IONPs at the concentration of 200 µg/mL for 4 h, respectively. Cells were washed with PBS, fixed with ice-cold acetone, stained with 2% potassium ferrocyanide II/1 M hydrochloric acid mixture (1:1) for 10 min at 37 °C, and subsequently counterstained with nuclear fast red solution (0.1%, w/v) for 1 min^28^.

#### ICP-MS measurement

RAW264.7 macrophages were treated with IONPs with different concentrations (100 and 500 μg/mL) for different incubation time (1, 2 and 4 h) at 37 °C, respectively. Cells were washed with PBS, trypsinized, and digested with aqua regia (a 3:1 mixture of hydrochloric acid and nitric acid) at 70 °C. The iron concentration was determined by inductively coupled plasma mass spectrometry (ICP-MS, Agilent 7500, Santa Clara, CA, USA).

#### Particle location by TEM

RAW264.7 macrophages and SKOV-3 cells were incubated with IONPs at the concentration of 100 µg/mL for 2 h. Cells were fixed with 2% paraformaldehyde and 1% glutaraldehyde, dehydrated in graded dilutions of ethanol, embedded in artificial resin and processed for electron microscopy. Subcellular localization of IONPs was monitored by Philips CM-120 TEM.

### Cytotoxicity assays

#### MTS assay

RAW264.7 macrophages and SKOV-3 cells were incubated with IONPs of different concentrations for 48 h. The cytotoxicity of IONPs was measured by MTS assay. Cell viability was expressed as the percentage of viable cells in the treated group compared to the untreated controls.

#### Hoechst 33342 and PI staining assay

SKOV-3 cells were incubated with SEI-10 (5 µg/mL), SMG-10 (400 µg/mL), or SMG-30 (400 µg/mL) for 16 h, and then stained with Hoechst 33342 (1 µg/mL) and PI (5 µg/mL) for 15 min at 37 °C. Stained nuclei were observed under fluorescence microscopy (Olympus IX83).

#### Lactate dehydrogenase leakage assay

SKOV-3 cells were incubated with IONPs of different concentrations for 4 h at 37 °C. 50 μL of the supernatant was transferred to a new 96 well plate, and cells were lysed with 50 μL 2% Triton-X100 for 30 min at 37 °C. 50 μL of reconstituted substrate was added to both the supernatant and the cell lysate plates. Optical density (OD) was measured at 492 nm using a microplate reader. LDH leakage was calculated using the following equation: [Supernatant OD/(Supernatant OD + Lysate OD) × 100]^29^.

#### Hemolysis

Red blood cells (RBCs) were prepared using freshly harvested mouse blood. 200 μL of RBCs suspension (2% in PBS) was mixed with different concentrations of IONPs and incubated at 37 °C for 4 h. Following the incubation, the tubes were centrifuged at 1000 × g for 5 min, and the supernatant was collected for the detection of free hemoglobin at the absorbance of 540 nm. PBS was used as a negative control and Triton-100 (2%) was uses as a positive control. The percent hemolysis was calculated using the following formula: (OD _sample_ − OD _negative control_)/(OD _positive control_ − OD _negative control_) × 100%.

#### Annexin V-FITC/PI staining

SKOV-3 cells were treated with IONPs at indicated concentrations for 24 h. Then, cells were washed with PBS, harvested, stained with 5 μL Annexin V-FITC solution and 5 μL PI solution for 30 minutes in dark, and then analyzed with a Becton Dickinson LSR II flow cytometer (BD Bioscience, San Jose, CA, USA)^30^. 10,000 events were recorded for each flow cytometric analysis.

#### Measurement of Intracellular ROS

Reactive oxygen species (ROS) were measured by the DCF-DA assay as described previously^31^. SKOV-3 cells were incubated with IONPs at indicated concentrations for 18 h. Then, cells were stained with 10 μM DCF-DA in a dark and humidified atmosphere (5% CO_2_, 37 °C) for 30 min. ROS production (green fluorescence) was either qualitatively observed by confocal fluorescence microscopy (Olympus IX83), or quantitatively analyzed by flow cytometry (Ex at 488 nm, Em at 525 nm).

#### Mitochondrial membrane potential (MMP)

SKOV-3 cells incubated in 6-well plates were subjected to the exposure of INOPs at the indicated concentrations for 18 h. Cells were incubated with 40 nM DiOC6(3) for 30 min, harvested, washed three times with PBS, and then analyzed by flow cytometry.

#### Cell cycle analysis

SKOV-3 Cells were treated with IONPs at indicated concentrations at 37 °C for 24 h. Cells were harvest, washed with PBS, fixed in 75% ethanol overnight at 4 °C. Then, cells were washed with PBS, treated with ribonuclease (RNase, 100 μg/mL), and stained with PI (10 μg/mL) for 30 min in dark. The samples were analyzed by flow cytometry under a 543-nm argon ion laser. Untreated cells were used as negative controls. Approximately 1 × 10^4^ counts were collected for each sample.

#### Western blot

SKOV-3 cells were incubated with IONPs at indicated concentrations for 18 h. Cells were then washed with ice-cold PBS and lysed in RIPA buffer. Samples were run on sodium dodecyl sulfate-polyacrylamide gels (SDS-PAGEs), transferred onto PVDF membranes, and incubated with appropriate primary antibodies (Bcl-2, Bax, Cyclin D, LC3B, and actin). Blots were then incubated with horseradish peroxidase (HRP)-conjugated secondary antibodies and visualized by enhanced chemiluminescence (GE Healthcare, Little Chalfont, Buckinghamshire, UK).

#### *In vivo* biodistribution

Female nude mice were injected subcutaneously with SKOV-3 cells (5 × 10^6^ cells in PBS) on the right flank to establish ovarian cancer xenograft mouse model. When the tumors size reached an approximate diameter of 8–10 mm, the mice were randomly divided into four groups, with 4 mice in each group. Tumor bearing mice were intravenously injected with PBS or various IONPs (1.5 mgFe/kg), respectively. At 24 h post-injection, tumor tissues, blood and main organs (liver, spleen, lung, heart, and kidney) were collected, weighed and then digested in aqua regia. The iron concentration in the samples was determined by ICP-MS.

To evaluate their biodegradation and clearance, various IONPs were intravenously injected into BLAB/c mice at a dose of 1.5 mgFe/kg (n = 4). Liver and spleen were collected at 6 h and 2 weeks after injection, fixed in 10% neutral buffered formalin, embedded in paraffin, cut into 10 μm-thick slices. The sections were processed with Prussian blue and nuclear fast red staining.

#### *In vivo* toxicity

BALB/c mice (n = 4) were intravenously injected with various IONPs at different dose (1.5, 2.5, or 5 mg/kg), respectively. The body weight of mice was weighed twice per week for 2 weeks. On day 7, blood samples were obtained from all mice for hematological and serum chemistry analysis, including complete blood count (CBC), alanine aminotransferase (ALT), aspartate aminotransferase (AST), total bilirubin, blood urea nitrogen (BUN) and creatinine. After 2 weeks, the mice were sacrificed, and liver and spleen tissues were harvested for histological analysis. The sections were stained with hematoxylin and eosin and then examined by a pathologist.

### Statistical analysis

Data was analyzed in GraphPad Prism (version 6.01) by one-way analysis of variance (ANOVA) followed by Dunnett’s multiple comparison, respectively. *P* values lower than 0.05 were considered statistically significant. Data are expressed as the mean ± standard deviation (SD).

### Data availability

No datasets were generated or analyzed during the current study.

## Electronic supplementary material

Supplementary Information

## References

[1] Naqvi S (2010). Concentration-dependent toxicity of iron oxide nanoparticles mediated by increased oxidative stress. Int. J. Nanomedicine.

[2] Bobo D, Robinson KJ, Islam J, Thurecht KJ, Corrie SR (2016). Nanoparticle-Based Medicines: A Review of FDA-Approved Materials and Clinical Trials to Date. Pharm. Res..

[3] Bulte JW (2009). *In vivo* MRI cell tracking: clinical studies. AJR.American J. Roentgenol..

[4] Wang Y-XJ (2015). Current status of superparamagnetic iron oxide contrast agents for liver magnetic resonance imaging. World J Gastroenterol.

[5] Gustafson HH, Holt-Casper D, Grainger DW, Ghandehari H, Grainger D (2015). Nanoparticle Uptake: The Phagocyte Problem HHS Public Access. Nano Today.

[6] Kumar A, Pandey AK, Singh SS, Shanker R, Dhawan A (2011). Cellular uptake and mutagenic potential of metal oxide nanoparticles in bacterial cells. Chemosphere.

[7] Singh R (2006). Tissue biodistribution and blood clearance rates of intravenously administered carbon nanotube radiotracers. Proc. Natl. Acad. Sci. USA.

[8] Lechanteur A (2016). PEGylation of lipoplexes: The right balance between cytotoxicity and siRNA effectiveness. Eur. J. Pharm. Sci..

[9] Nam K, Jung S, Nam J, Kim SW (2015). Poly (ethylenimine) conjugated bioreducible dendrimer for ef fi cient gene delivery. J. Control. Release.

[10] Shen J (2014). Cyclodextrin and polyethylenimine functionalized mesoporous silica nanoparticles for delivery of siRNA cancer therapeutics. Theranostics.

[11] Xiao K (2011). The effect of surface charge on *in vivo* biodistribution of PEG-oligocholic acid based micellar nanoparticles. Biomaterials.

[12] Schweiger C (2012). Quantification of the internalization patterns of superparamagnetic iron oxide nanoparticles with opposite charge. J. Nanobiotechnology.

[13] Enochs WS, Harsh G, Hochberg F, Weissleder R (1999). Improved delineation of human brain tumors on MR images using a long-circulating, superparamagnetic iron oxide agent (COMBIDEX***). J. Magn. Reson. Imaging.

[14] Moore A, Marecos E, Bogdanov A, Weissleder R (2000). Tumoral Distribution of Iron Oxide Nanoparticles in a Rodent Model. Radiology.

[15] Zimmer C (1997). Tumor Cell Endocytosis Imaging Facilitates Delineation of the Glioma–Brain Interface. Exp. Neurol..

[16] Zimmer C (1995). MR imaging of phagocytosis in experimental gliomas. Radiology.

[17] Mcneil SE (2005). Nanotechnology for the biologist. J. Leukoc. Biol..

[18] Zhu, C. *et al*. Synthesis of novel galactose functionalized gold nanoparticles and its radiosensitizing mechanism. *J. Nanobiotechnology* 1–11 10.1186/s12951-015-0129-x (2015). 10.1186/s12951-015-0129-x PMC4600275 26452535

[19] Cortez MA (2015). The Synthesis of Cyclic Poly(ethylene imine) and Exact Linear Analogues: An Evaluation of Gene Delivery Comparing Polymer Architectures. J. Am. Chem. Soc..

[20] Negoda A, Kim K, Crandall ED, Worden RM (2013). Biochimica et Biophysica Acta Polystyrene nanoparticle exposure induces ion-selective pores in lipid bilayers. BBA - Biomembr..

[21] Liu YY (2014). Genotoxicity assessment of magnetic iron oxide nanoparticles with different particle sizes and surface coatings. Nanotechnology.

[22] Könczöl M (2011). Cytotoxicity and genotoxicity of size-fractionated iron oxide (magnetite) in A549 human lung epithelial cells: Role of ROS, JNK, and NF-κB. Chem. Res. Toxicol..

[23] Dreaden EC (2012). Small Molecule–Gold Nanorod Conjugates Selectively Target and Induce Macrophage Cytotoxicity towards Breast Cancer Cells. Small.

[24] Moghimi S (2005). A two-stage poly(ethylenimine)-mediated cytotoxicity: Implications for gene transfer/therapy. Molecular therapy: the journal of the American Society of Gene Therapy.

[25] Du J, Zhu W, Yang L, Wu C (2016). Reduction of polyethylenimine-coated iron oxide nanoparticles induced autophagy and cytotoxicity by lactosylation. Regen Biomter.

[26] McConnell K (2016). Reduced Cationic Nanoparticle Cytotoxicity Based on Serum Masking of Surface Potential. J. Biomed. Nanotechnol..

[27] Bao Y (2015). Magnetic Nanoparticles: Material Engineering and Emerging Applications in Lithography and Biomedicine. Journal of Materials Science.

[28] Schlorf T (2010). Biological properties of iron oxide nanoparticles for cellular and molecular magnetic resonance imaging. Int. J. Mol. Sci..

[29] Hanini A (2011). Evaluation of iron oxide nanoparticle biocompatibility. Int. J. Nanomedicine.

[30] Kuo, K. *et al*. MLN4924, a novel protein neddylation inhibitor, suppresses proliferation and migration of human urothelial carcinoma: *In vitro* and *in vivo* studies. *Cancer Lett*. 1–10, 10.1016/j.canlet.2015.01.015 (2015). 10.1016/j.canlet.2015.01.015 25615422

[31] Lebel CP, Ischiropoulos H, Bondys SC (1992). Evaluation of the Probe 2′,7′-Dichiorofluorescin as an Indicator of Reactive Oxygen Species Formation and Oxidative Stress. Chem. Res. Toxicol.

