# Supplementary material for: Uptake, distribution, clearance, and toxicity of iron oxide nanoparticles with different sizes and coatings
Source: Sci Rep. 2018 Feb 1;8:2082. doi: 10.1038/s41598-018-19628-z (PMC5794763; doi:10.1038/s41598-018-19628-z)
Supplement: Supplementary file 1 — Supplementary Information [file 41598_2018_19628_MOESM1_ESM.doc]

**Appendix A. Supplementary data**

Uptake, distribution, clearance, and toxicity of iron oxide nanoparticles with different sizes and coatings

Qiyi Feng^a,1^, Yanping Liu^b,1^, Jian Huang^c,e,1^, Ke Chen^a^ , Jinxing Huang^a^, and Kai Xiao^a,d^

^a^*National Chengdu Center for Safety Evaluation of Drugs, State Key Laboratory of Biotherapy, Collaborative Innovation Center for Biotherapy, West China Hospital, Sichuan University, Chengdu , China*

^b^*Safety Evaluation Center, Sichuan Institute for Food and Drug Control, Chengdu, China*

^c^*Department of Thoracic Surgery, West China Hospital, Sichuan University, Chengdu, China*

^d^*Laboratory of Non-Human Primate Disease Model research, State Key Laboratory of Biotherapy, Collaborative Innovation Center for Biotherapy, West China Hospital, Sichuan University, Chengdu, China*

*^e^Department of Thoracic Surgery, Wuxi People’s Hospital of Nanjing Medical University, Wuxi, China*


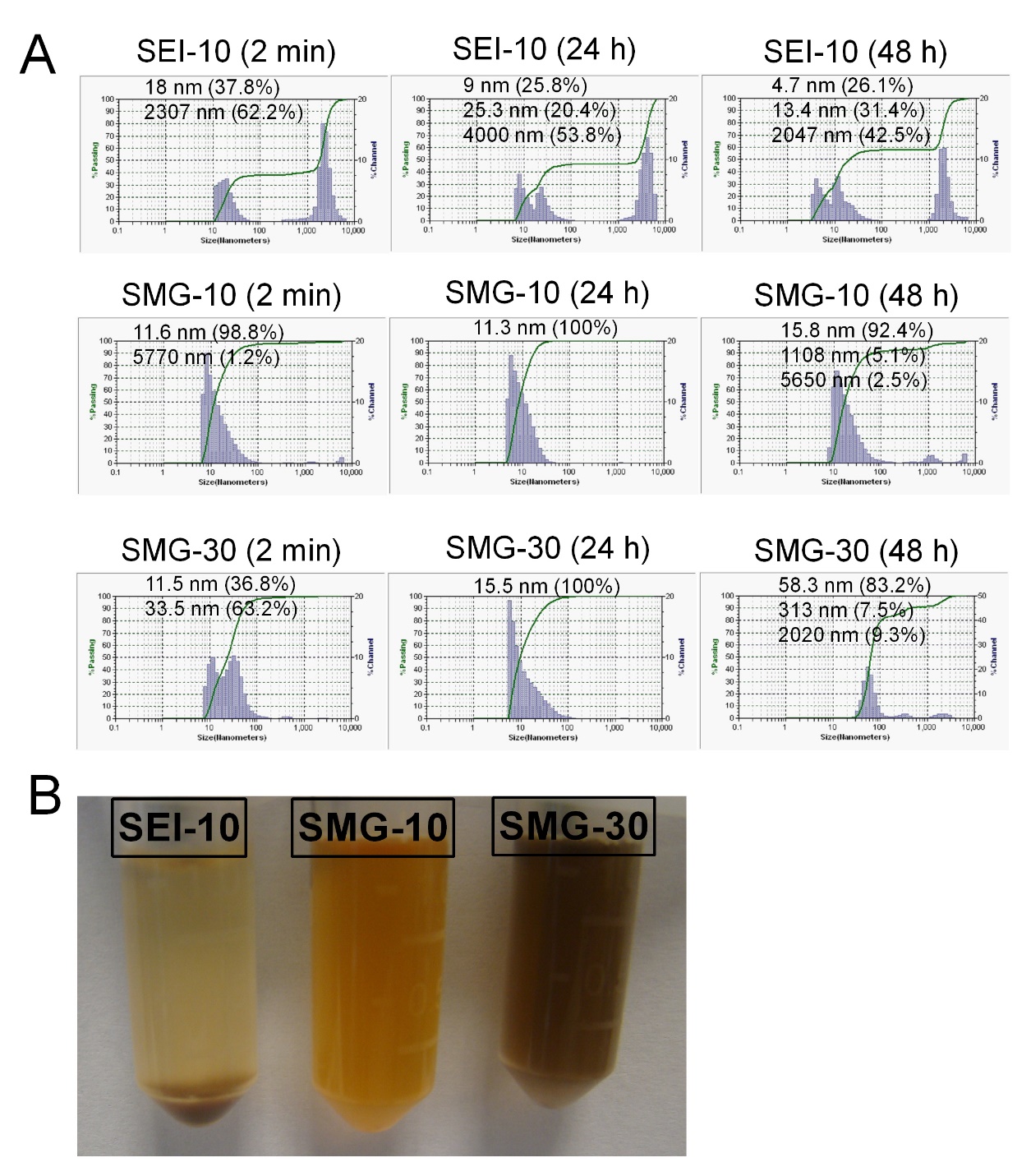


Supplemental Figure 1.

The stability of different IONPs in 50% human plasma. (A)The particle size change of IONPs in human plasma was monitored by DLS using Microtrac. (B) Representative pictures of IONPs in human plasma after 48-h incubation.


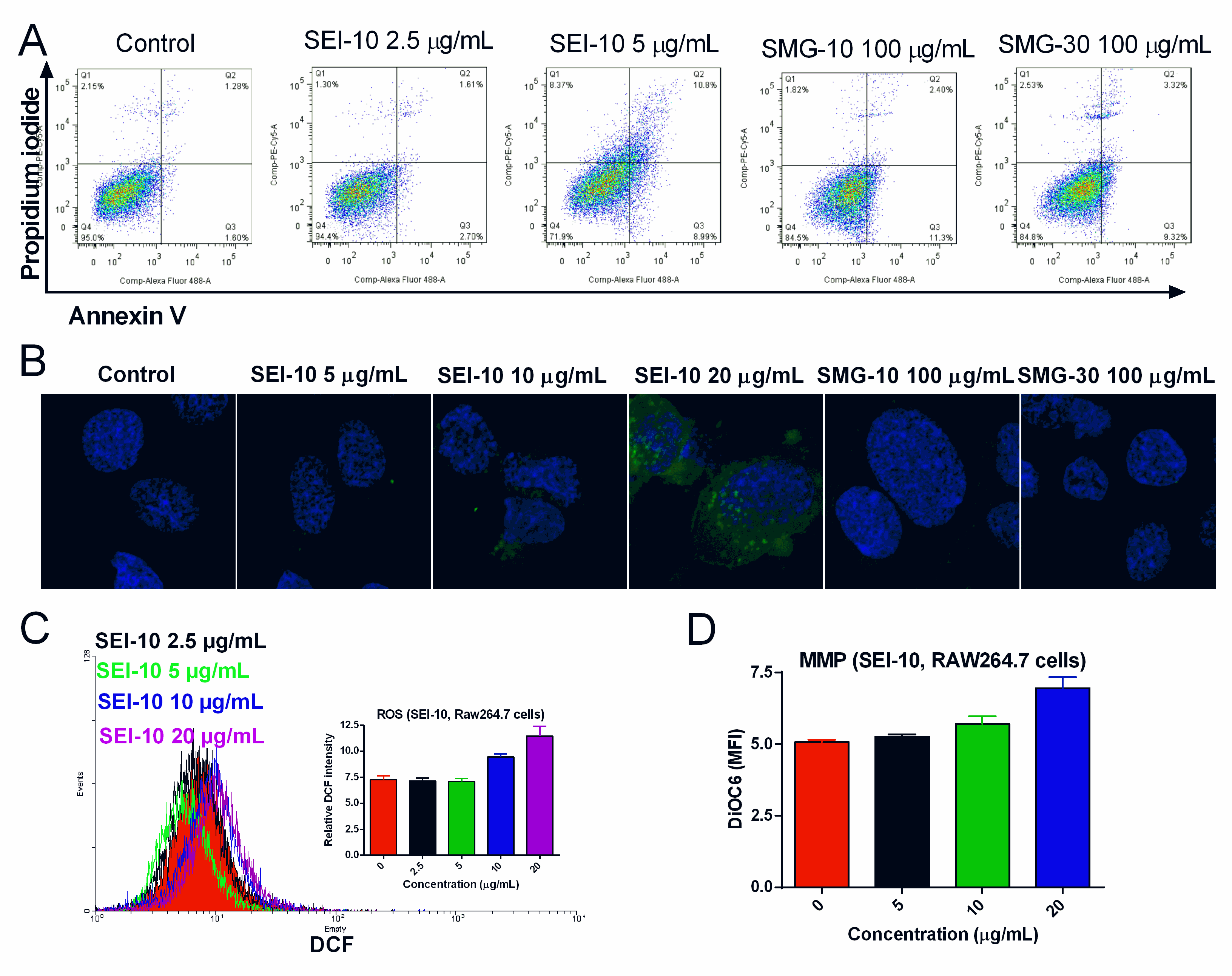


Supplemental Figure 2.

The potential mechanisms underlying the cell death induced by IONPs in RAW264.7 macrophages. (A) Apoptotic and necrotic cell death were analyzed by Annexin V and PI dual staining after IONPs treatment for 24 h in Raw264.7 macrophages. Intracellular reactive oxygen species (ROS) production in Raw264.7 macrophages treated with various IONPs for 18 h, was detected using a H2DCFDA probe by confocal microscopy (B) and flow cytometry (C), respectively. (D) The measurement of membrane mitochondria potential (MMP) of Raw264.7 macrophages treated with different concentrations of SEI-10 for 18 h.





Supplemental Figure 3.

The production of ROS in SKOV-3 cells after 18-h treatment of SMG-10 (A) and SMG-30 (B), was detected by flow cytometry using a H2DCFDA probe, respectively.

| 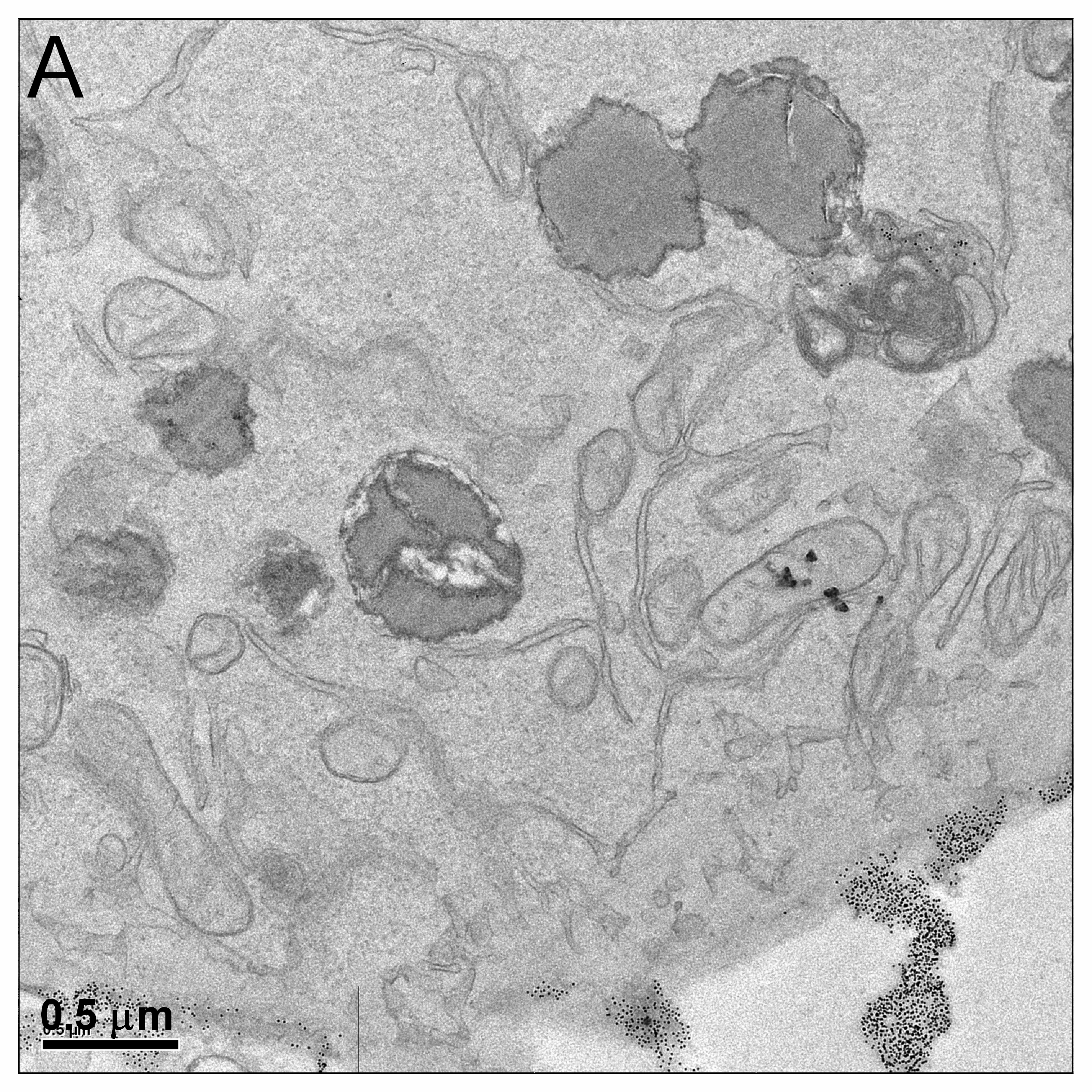 | 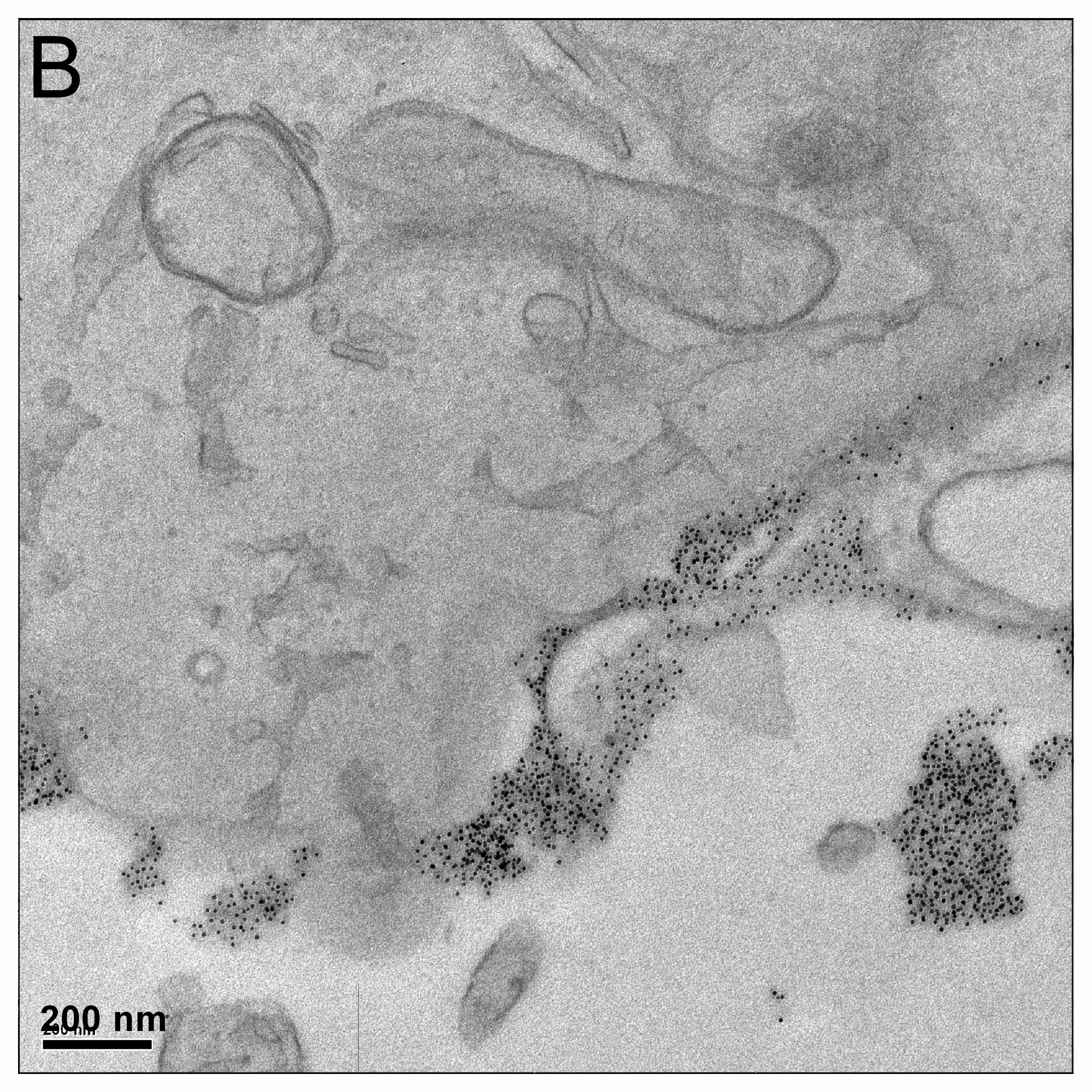 | 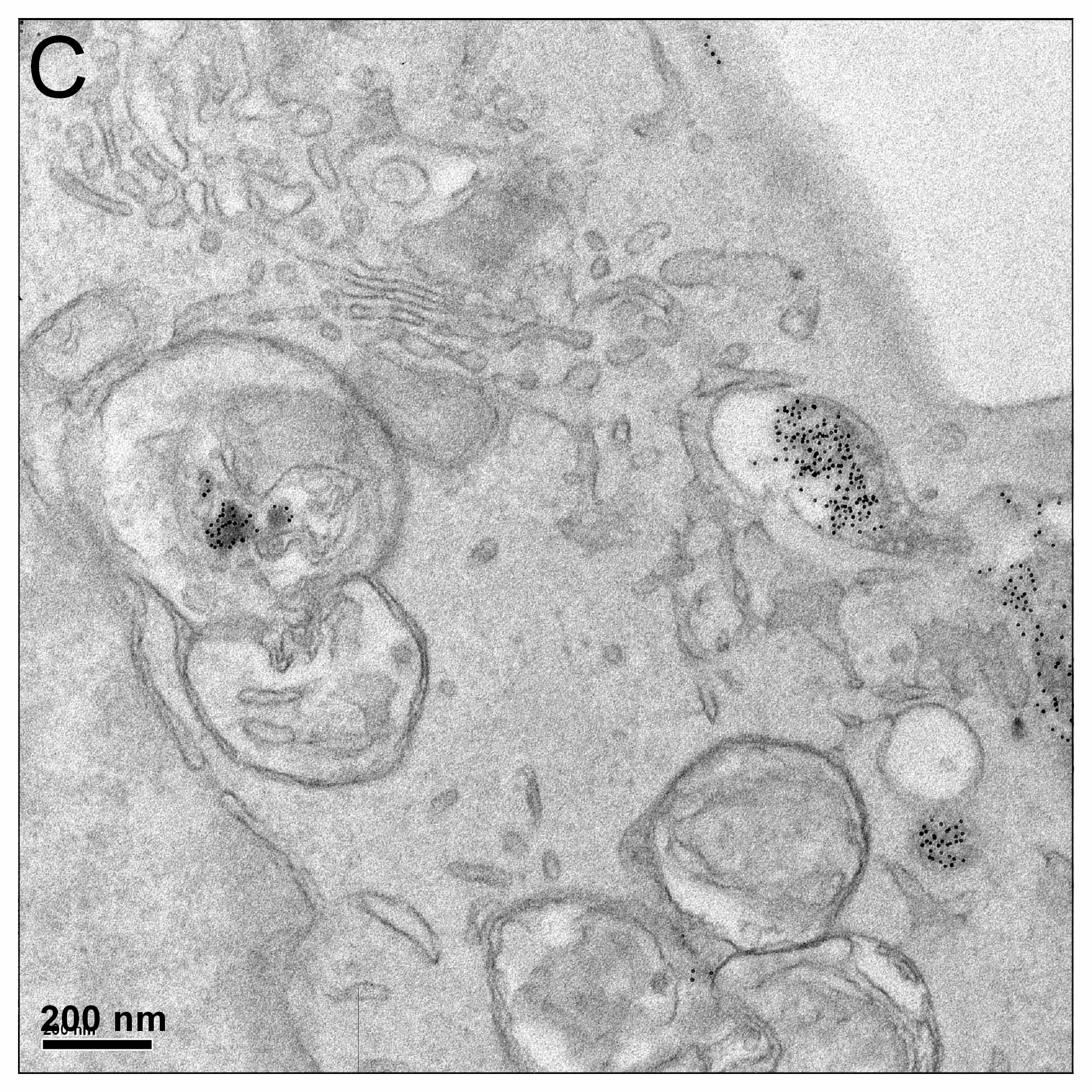 |
| --- | --- | --- |

Supplemental Figure 4.

TEM images showed the adsorptive endocytosis process of SEI-10 in SKOV-3 cells.

|  |  |
| --- | --- |
|  |  |

Supplemental Figure 5.

Relative densitometric analysis of protein expression in western blot experiments as shown in Figure 6F. The mean normalized optical density of Bcl, Bax, Cyclin D, and LC3B-Ⅱ protein bands relative to that of Actin bands from the same sample was calculated. Expression levels of these proteins are expressed as fold changes compared with the control group. Error bars represent mean ± SD (n = 3). **P*<0.05 vs. the control group.

| 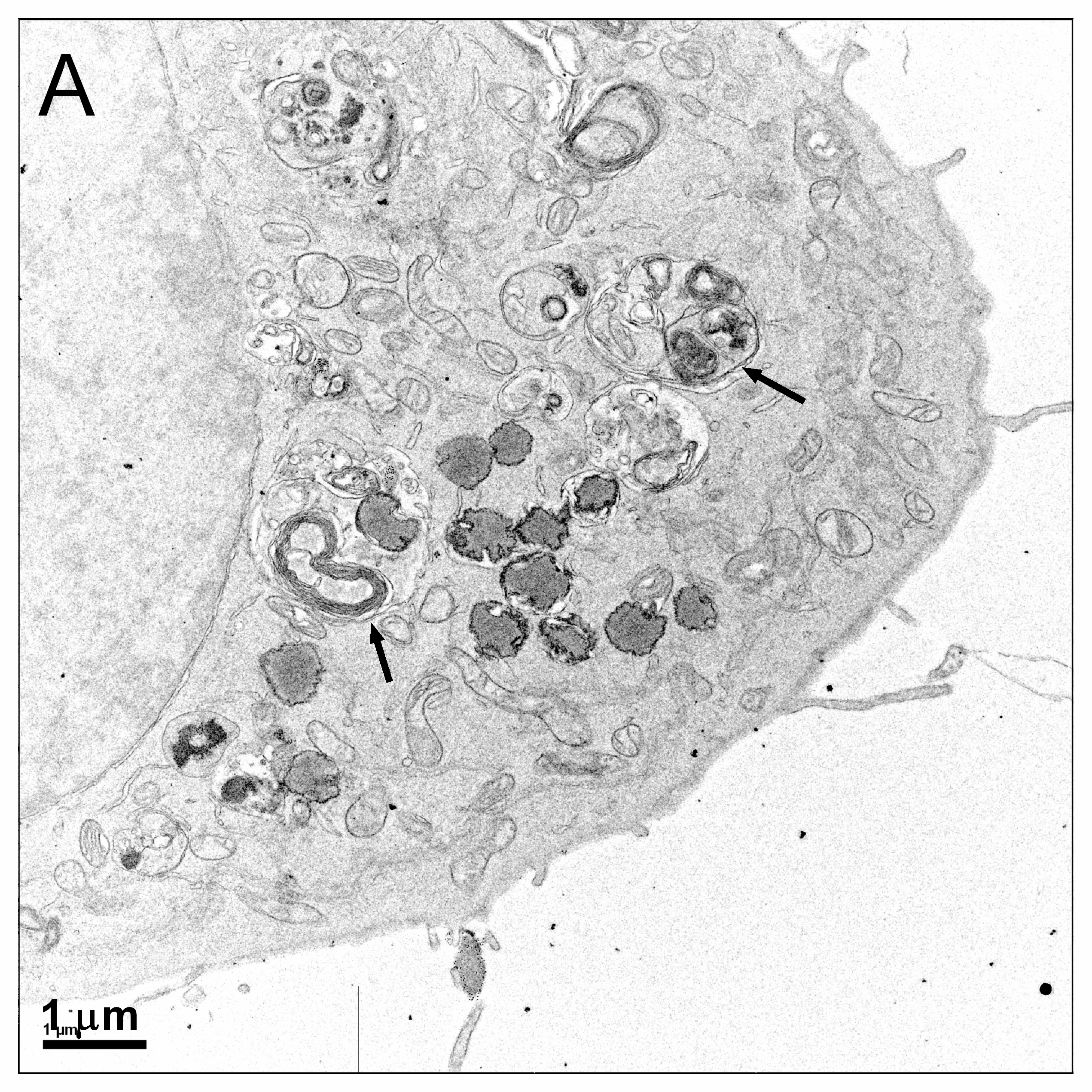 | 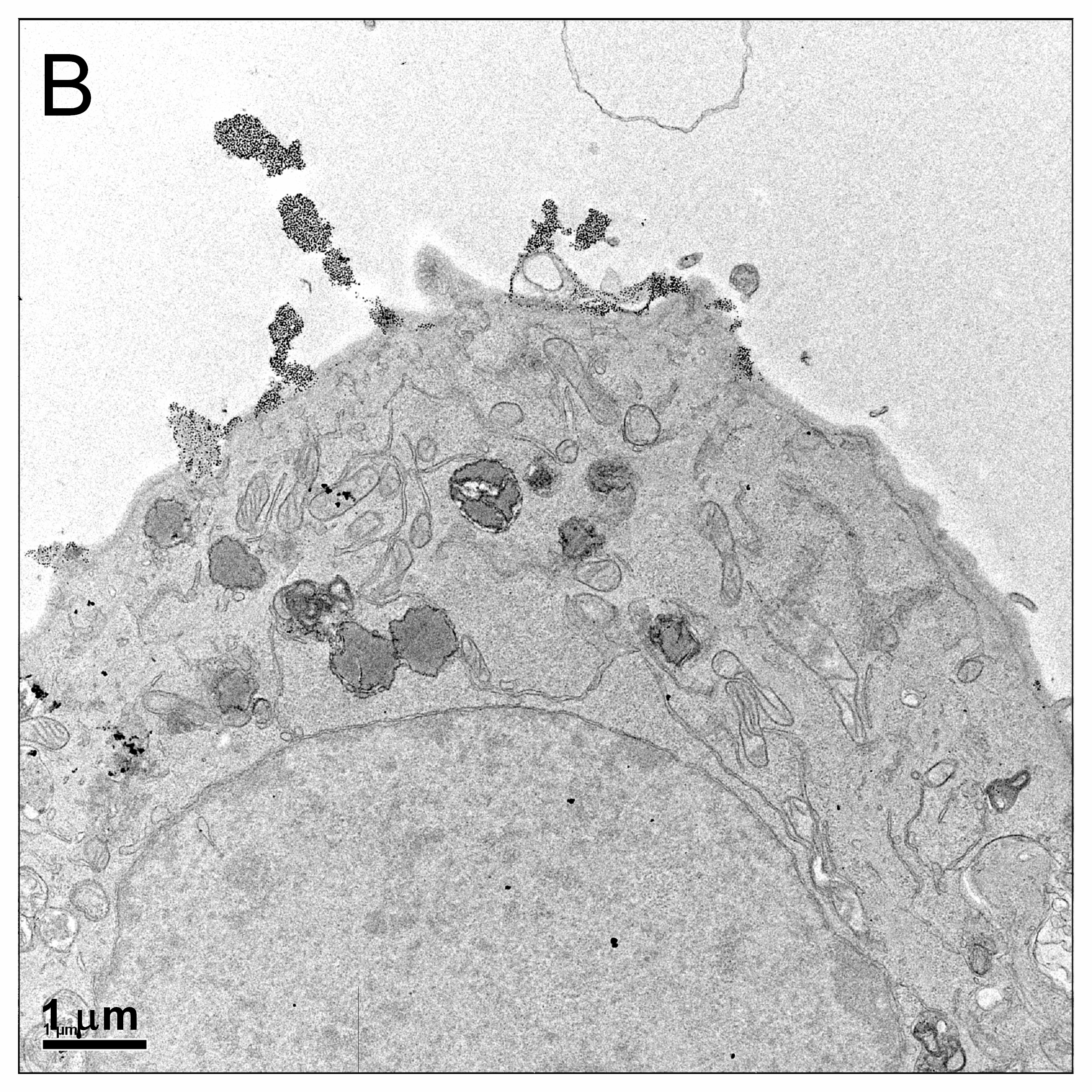 |
| --- | --- |

Supplemental Figure 6.

Representative TEM images verified the intracellular autophagosomes (black arrow) in SMG-10 treated cells (A), not in SEI-10 treated cells (B).

Supplemental Table 1.

Blood cell counts in mice on day 7 post-injection (1.5 mg/kg)

| Groups | WBC  (K/uL) | Absolute Neutrophil  (K/ul) | RBC  (M/uL) | Hemoglobin  (g/dL) | Platelets  (K/uL) |
| --- | --- | --- | --- | --- | --- |
| Control | 5.8 ± 2.0 | 1.7 ± 1.1 | 9.2 ± 0.5 | 14.4 ± 0.7 | 1069.8 ± 277.3 |
| SEI-10 | 7.4 ± 2.5 | 1.4 ± 0.3 | 9.0 ± 0.9 | 14.3 ± 0.7 | 1084.3 ± 326.1 |
| SMG-10 | 5.7 ± 2.4 | 1.3 ± 0.3 | 9.1 ± 0.5 | 14.5 ± 0.7 | 898.5 ± 158.1 |
| SMG-30 | 5.7 ± 0.8 | 1.0 ± 0.1 | 8.9 ± 0.5 | 13.8 ± 0.9 | 1004.0 ± 81.5 |

Supplemental Table 2.

Serum chemistry in mice on day 7 post-injection (1.5 mg/kg)

| Groups | ALT  ( U/L) | AST  ( U/L) | Total Bilirubin  (mg/dL) | BUN  (mg/dL) | Creatinine  (mg/dL) |
| --- | --- | --- | --- | --- | --- |
| Control | 25.2 ± 2.3 | 87.5 ± 13.3 | 0.083 ± 0.045 | 20.7 ± 4.4 | 0.074 ± 0.014 |
| SEI-10 | 30.8 ± 8.5 | 118.4 ± 70.7 | 0.076 ± 0.012 | 16.1 ± 4.1 | 0.084 ± 0.008 |
| SMG-10 | 39.2 ± 4.2* | 110.6 ± 12.5 | 0.063± 0.009 | 15.9 ± 3.0 | 0.085± 0.004 |
| SMG-30 | 28.1 ± 5.3 | 103.8 ± 14.9 | 0.066± 0.018 | 18.6 ± 5.4 | 0.074± 0.014 |

Note: **P*≤0.05, when compared to negative control.
